# Preclinical and clinical examinations of *Salvia miltiorrhiza *and its tanshinones in ischemic conditions

**DOI:** 10.1186/1749-8546-1-3

**Published:** 2006-11-23

**Authors:** James David Adams, Rubin Wang, Jun Yang, Eric Jungchi Lien

**Affiliations:** 1 Department of Molecular Pharmacology and Toxicology, School of Pharmacy, University of Southern California,1985 Zonal Avenue, Los Angeles, CA 90089-9121, USA; 2 Department of Pharmaceutical Sciences, School of Pharmacy, University of Southern California, 1985 Zonal Avenue, Los Angeles, CA 90089-9121, USA; 3 Department of Psychiatry and Behavioral Sciences, School of Medicine, University of Southern California, Los Angeles, CA 90089-9151, USA

## Abstract

*Salvia miltiorrhiza *(*Labiatae*, *Laminaceae*), *danshen*, is an annual sage mainly found in China and neighboring countries. The crude drug (dried root) and its preparations are currently used in China to treat patients suffering from heart attack, angina pectoris, stroke and some other conditions. The use of *S. miltiorrhiza *has been increasing in the management of stroke. Pharmacological examinations showed that the plant and its active ingredients, tanshinones and salvianolic acids, have anticoagulant, vasodilatory, increased blood flow, anti-inflammatory, free radical scavenging, mitochondrial protective and other activities. This review discusses the pharmacology, medicinal chemistry and clinical studies published, especially in China, for *danshen *and tanshinone preparations. Clinical examinations are evaluated in terms of *S. miltiorrhiza *preparation, dose, double blinding, control, clinical assessments of outcomes and other parameters. Meta-analyses of *S. miltiorrhiza *are also discussed.

## Background

*Danshen *is an annual sage plant, *Salvia miltiorrhiza *(*Labiatae*, *Laminaceae*) which grows in China, Mongolia, Korea and Japan. In China, it is found in hilly areas of the west, southwest and southeast. *S. miltiorrhiza *is among the most popular medicinal herbs used in China. It has been used in the treatment of stroke since 1970 [1], angina and heart attack, as an antihypertensive and a sedative [2]. *S. miltiorrhiza *contains several compounds that are pharmacologically active, especially the diterpenoids known as tanshinones. A related plant, *Salvia columbariae*, from California, USA also contains tanshinones, especially cryptotanshinone [3]. This plant has been used by Californian Indians to treat people suffering from strokes [4].

*S. miltiorrhiza *is often used in *fufang *with other herbs in Chinese herbal medicine. *Fufang *in Chinese herbal medicine means a formula comprising multiple herbs. One of the widely prescribed Chinese *fufang *for stroke patients is 30 g of the roots *of Astragalus membranaceus *and *Hedysarum coronarium*, 10 g of cinnamon twig (*Cinnamomum verum*), 10 g of peach kernel (*Prunus persica*), 10 g of safflower (*Carthamus tinctorius*), 12 g *of Angelica sinensis *root, 12 g of the rhizome of *Ligustica chuanxiong*, 12 g of the root of *Paeonia rubra*, 12 g of earthworm (*Lumbricus*), 30 g of the root of *S. miltiorrhiza *and 15 g of the root of *Achyranthis bidentata *[5]. The ingredients are added to water and boiled for an hour or two until the required volume is attained. The preparation is orally administered to the patient. Other plants can be added to this as needed by the patient for severe blood stasis, deficiency of *qi *and some other conditions. *Qi *is the life force that comes from air, food and the genetic background. The therapeutic principle of the treatment is to supplement *qi*, nourish the blood and promote blood circulation in order to remove obstructions of the channels, which are the acupuncture channels, not blood vessels.

## Pharmacology

### Danshen

*S. miltiorrhiza *has been shown to inhibit angiotensin converting enzyme (ACE), to lower blood pressure, dilate arteries, and to decrease blood clotting [6-9]. Inhibition of ACE may be involved in the ability of *S. miltiorrhiza *to alter angiotensin II levels, and indirectly, the levels of atrial natriuretic peptide [10,11]. ACE inhibitors have been extensively studied in cardiovascular disease and have been shown in several clinical trials to decrease the risk of suffering a primary or secondary stroke [12-14]. ACE inhibitors are known to decrease the risk of having a stroke. Unfortunately, Western ACE inhibitor drugs have not been tested as treatments for stroke after its occurrence. The primary mechanism of this protection appears to be in lowering blood pressure. However, other mechanisms are also probably involved including decreasing clot formation by decreasing cardiac fibrillation, and decreasing the levels of angiotensin. Angiotensin has several potentially dangerous effects in regard to stroke, including hypertension, increasing noradrenergic nerve activity and blocking norepinephrine reuptake in the brain [15]. Brain ischemia and reperfusion, during stroke, cause the release of many neurotransmitters including norepinephrine, in part due to ATP depletion. These neurotransmitters may be damaging to the brain by inducing excitotoxicity, by oxidizing to produce oxygen radicals or other mechanisms. The effects of angiotensin on extracellular neurotransmitter levels during ischemia and reperfusion in the brain could be devastating. *S. miltiorrhiza *has been shown to decrease the release of norepinephrine, dopamine and serotonin during brain ischemia [16].

Thrombosis secondary to atherosclerosis is a major cause of stroke. Small thrombi lodge in small arteries of the brain, especially the middle cerebral artery, and cause ischemia. Fibrinolytic enzymes normally found in the blood such as plasmin, break down clots within a few hours and allow reperfusion. Clot dissolution with tissue plasminogen activator is known to be beneficial in some stroke patients, provided that it is used within 3 hours of stroke. Inhibition of clot formation and potential clot dissolution has been demonstrated in many clinical trials of *S. miltiorrhiza *as discussed below. *S. miltiorrhiza *has been found to increase the proteolysis of fibrinogen to fibrinogen degradation products [17]. This is a unique mechanism of anticoagulation in comparison to other anticoagulant drugs that tend to prevent clot formation by interfering with platelet function or interfering with the action of thrombin or vitamin K. *S. miltiorrhiza *induced arterial dilation in the brain could help reperfuse the brain better allowing a faster recovery. Ischemia causes ATP and NAD levels to decrease in the brain [18]. Reperfusion allows oxygen to reflow into the brain and form reactive oxygen radicals, especially when mitochondrial and cellular enzymes are not functioning efficiently due to low oxygen tensions [19]. These oxygen radicals can damage cellular macromolecules, including DNA, and induce apoptosis and necrosis [20].

*S. miltiorrhiza *has other effects on stroke including anti-inflammation, free radical scavenging, antioxidant and mitochondrial protection effects. Tanshinone I from *S. miltiorrhiza *inhibits arachidonic acid metabolism, interleukin-12 production and has anti-inflammatory effects [21,22]. Neutrophil activation is inhibited by an unspecified tanshinone [23] which is an anti-inflammatory agent. *S. miltiorrhiza *antioxidant activity is also expressed as increases in superoxide dismutase, catalase, glutathione peroxidase and glutathione transferase activities [24,25]. Free radical scavenging and mitochondrial protective activities of *S. miltiorrhiza *have been found [26-28].

### Fufang danshen

Clinical trials demonstrated that *fufang danshen *enhanced stroke survival and recovery in comparison to *S. miltiorrhiza *alone as discussed below. The idea is that additional herbs will synergize the desirable effects or decrease the side effects of *S. miltiorrhiza*. Commercially available *fufang danshen *can be in two forms, one containing *S. miltiorrhiza *and *jiangxiang *(*Dalbergia odorifera*), the other containing *S. miltiorrhiza*, *Panax notoginseng *and *Ligusticum wallichii*. Most published clinical trials used the *Dalbergia odorifera *form of *fufang danshen*.

*Dalbergia odorifera *contains medicarpin and 6-hydroxy-2-(2-hydroxy-4-methoxyphenyl) benzofuran(IV) that inhibit leukotriene synthesis [29]. *Dalbergia odorifera *also contains anti-inflammatory flavonoids such as (S)-4-methoxydalbergione, cearoin, butein, koparin, bowdichione, 3'-O-methylviolanone and xenognosin B [30]. Platelet aggregation and prostaglandin synthesis are inhibited by cinnamylphenols, isoflavans, isoflavene and a benzoic acid derivative from *Dalbergia odorifera *[31]. Vasorelaxant compounds are found in *Dalbergia odorifera *such as butein [32] and isoliquiritigenin [33]. *Panax notoginseng*, which contains several ginsenosides and has anti-platelet activity [34], has been found to improve survival in patients of cerebral trauma and cerebral ischemia reperfusion [35,36]. *Ligusticum wallichii *contains tetramethylpyrazine that is protective in ischemia reperfusion injury of the brain [37], has anti-platelet activity [38] and is hypotensive perhaps due to calcium antagonism [39]. *Ligusticum wallichii *also contains ferulinolol that is a β_1 _blocker and a partial β_2 _agonist [40].

## Medicinal chemistry

As *S. miltiorrhiza*, especially *fufang danshen*, have many pharmacologically active compounds in the preparation, one would ask which compound is the active one for stroke and ischemic diseases. The answer is that many active compounds in the preparations are beneficial in stroke and ischemic diseases. Among all active compounds, diterpenoids and salvianolic acid derivatives are most studied.

**Figure 1:**
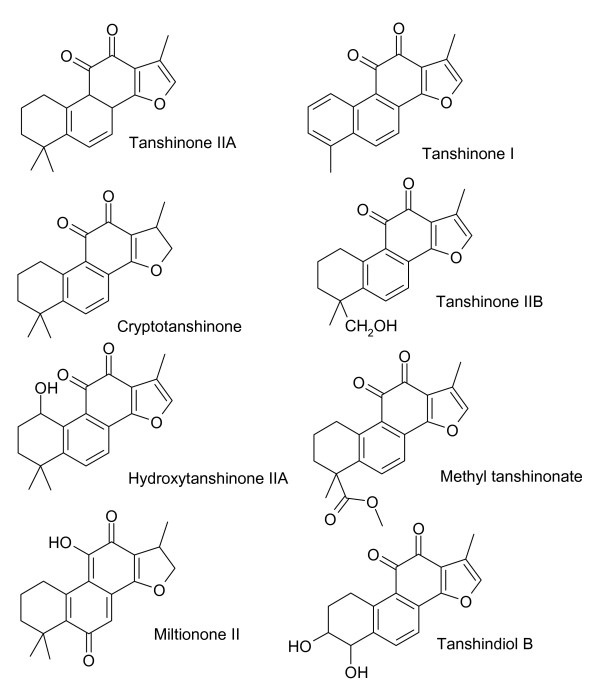
**Tanshinones found in *S. miltiorrhiza***. All of these compounds contain benzoquinone functionalities.

**Figure 2:**
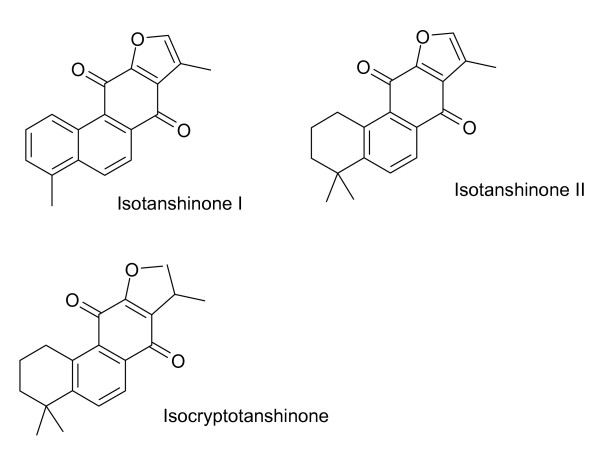
**Isotanshinones found in *S. miltiorrhiza***. All of these compounds contain naphthoquinone groups.

**Figure 3:**
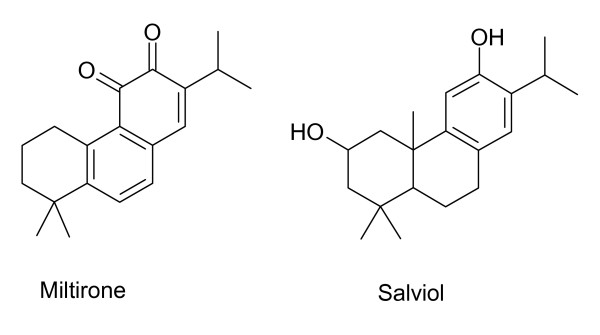
**Miltirone and salviol from *S. miltiorrhiza***. These compounds contain phenol or benzoquinone groups.

The diterpenoids of *S. miltiorrhiza *are characterized by tanshinones (Figure 1) and isotanshinones (Figure 2). Miltirone, salviol (Figure 3) and other diterpenoids are also present. Miltirone has sedative activity and is a benzodiazepine receptor agonist [41]. Purified tanshinone IIA and IIB are neuroprotective in cerebral ischemia and reperfusion [42]. Tanshinone I, cryptotanshinone and tanshinone V are protective against myocardial ischemia and reperfusion [2]. Tanshinones are also anti-inflammatory agents. Tanshinone I, dihydrotanshinone and cryptotanshinone inhibit interleukin-12 and interferon-γ production [22]. Tanshinone I inhibits arachidonic acid metabolism by phospholipase A_2 _[21].

**Figure 4:**
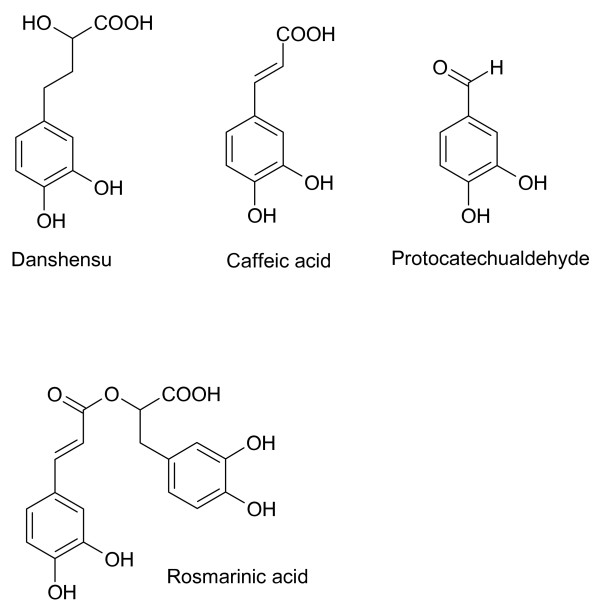
**Danshensu and monoterpenoids from *S. miltiorrhiza***. All of these compounds contain catechol functionalities.

**Figure 5:**
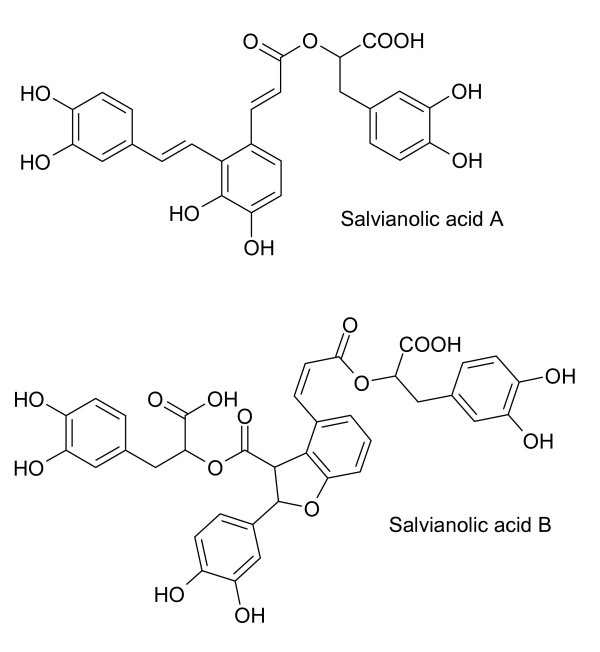
**Salvianolic acids from *S. miltiorrhiza***. All of these compounds contain catechol moieties.

**Figure 6:**
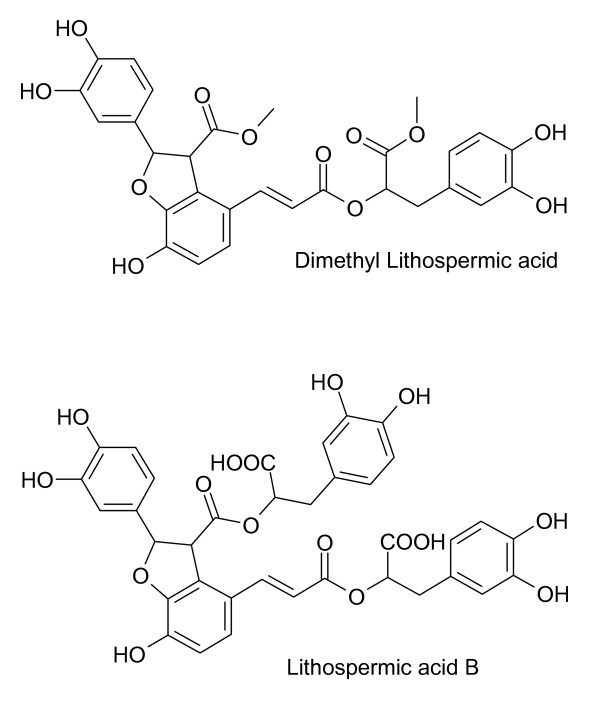
**Lithospermic acids from *S. miltiorrhiza***. Each of these compounds contains two or more catechols.

Salvianolic acids in *S. miltiorrhiza *appear to be synthesized from monoterpenoids such as *danshensu *(Figure 4). Acetylsalvianolic acid A, a semi-synthetic derivative of salvianolic acid from *S. miltiorrhiza *is neuroprotective in middle cerebral artery thrombosis [43] and inhibits platelet aggregation [44]. Salvianolic acids (Figure 5) from *S. miltiorrhiza *increase cerebral blood flow after ischemia [45]. Salvianolic acid A is protective against cerebral and myocardial ischemia and reperfusion [46]. Lithospermic acid B (Figure 6), also called tanshinoate B or salvianolic acid B, increases NO production by endothelial cells [47] and inhibits ACE [6]. NO is a vasorelaxant that should bring down local blood pressure. Lithospermic acid B is antihypertensive and is protective against cerebral and myocardial ischemia and reperfusion [46]. Rosmarinic acid and salvianolic acids in *S. miltiorrhiza *inhibit thrombosis, thromboxane B_2 _formation and platelet aggregation [45,46].

*Danshensu *literally means the active ingredient in *S. miltiorrhiza *and has been used to refer to different active compounds. Early investigators identified 2-hydroxy-4-catecholbutyric acid (Figure 4) in *S. miltiorrhiza*, assumed it was the active ingredient in *S. miltiorrhiza*, and named it *danshensu*. Later work identified tanshinone IIA as the primary active ingredient in the treatment of ischemic diseases. Most Chinese publications in the last 20 years refer to tanshinone IIA as *danshensu*. As the salvianolic acids in *S. miltiorrhiza *are also responsible for the effects of *S. miltiorrhiza *in ischemic disorders, salvianolic acid B has recently been referred to as *danshensu *as well by some Chinese manufacturers.

## Clinical trials

### A chronological analysis

*S. miltiorrhiza *has been tested in clinical trials of ischemic diseases such as angina, heart attack and stroke. These trials have been discussed in other publications [1,2,47-50]. Time of onset of stroke or myocardial infarction is a confounding issue in many of the trials. It is not unusual for clinical trials to begin treatment with *S. miltiorrhiza *1 week or more after an ischemic attack. Many Chinese patients were treated initially (within a few hours of onset) with the standard therapy for stroke or heart attack by local medical personnel before admission to a hospital. In most regions of China, the standard therapy for a heart attack or stroke is *S. miltiorrhiza*. The dosage of *S. miltiorrhiza *is usually clearly stated in published trials.

In a 1979 publication [51] of angina pectoris, 89 male and 19 female patients aged 40–75 were studied. Tanshinone IIA, as a sodium sulfonate salt, was injected intravenously (IV) in doses of 80 mg per day. The study was not blinded or randomized and had no controls. Symptoms and electrocardiograph (ECG) changes were monitored. The patients who responded to tanshinone IIA injections within 4 treatments or less gave an overall effective rate of 86%. The authors found that tanshinone IIA gave results similar to *fufang danshen*. Twelve patients with acute myocardial infarction were treated with tanshinone IIA, where improvement was found in ten cases. Tanshinone IIA produced a mild antihypertensive effect and decreased plasma viscosity.

Chen *et al*. [52] published a study of *fufang danshen*, as purified coronary heart II tablets, in 112 angina patients. The tablets contained extracts of *S. miltiorrhiza*, *Paeonia obovata*, *Ligusticum wallichii*, *Carthamus tinctorius *and *Dalbergia odorifera*. The doses of each ingredient were not stated. The study was randomized, double blinded and placebo controlled. Improvements were recorded in terms of clinical symptoms and ECG improvements. *S. miltiorrhiza *was 80% effective compared to a 16% effective rate for the placebo.

A study was published in 1991 concerning the use of *S. miltiorrhiza *in 120 stroke patients [53]. The study was randomized, but not blinded or placebo controlled. There were 96 male and 24 female patients between the ages of 50 and 84. The patients either had strokes (86 cases) or transient ischemic attacks (14 cases). They were administered *S. miltiorrhiza *containing 40 mg of tanshinone IIA in either a 40 ml injection or a 500 ml IV drip. The time of administration after the ischemic attack ranged from 3–10 hours in the transient ischemic attack cases, or 1–15 days in the stroke cases. The *S. miltiorrhiza *preparations were 88% effective in alleviating the symptoms of ischemic diseases. It was claimed that the 40 ml injection form was better than the 500 ml IV drip form of *S. miltiorrhiza*.

A study of subarachnoid bleeding in 42 patients was published in 1992 [54]. There were 18 male and 24 female patients. Two patients were younger than 50. Thirteen patients were between 51 and 60 years old. Twenty one patients were older than 61. The study was not randomized, blinded or placebo controlled. The time of administration of *S. miltiorrhiza *ranged from 3 to 7 days after onset. The *S. miltiorrhiza *group of 42 patients received IV *S. miltiorrhiza *containing 25 – 75 mg of tanshinone IIA, daily for 3 weeks. The control group of 38 patients received 24 g of 6-aminocaproic acid (a hemostatic agent) each day for 3 weeks. The intracranial pressure of each patient was monitored and corrected with 125 – 250 ml of 20% mannitol daily for 7 – 10 days. High intracranial pressure was above 23.51 kPa. Clinical symptoms and blood in the cerebrospinal fluid were assessed in each patient. *S. miltiorrhiza *was 95% effective compared to 79% efficacy in the control group. *S. miltiorrhiza *cleared hemoglobin from the cerebrospinal fluid within 3 weeks, whereas 4 weeks were required in the control group.

*Fufang danshen *containing *Dalbergia odorifera *was used in a study of 95 patients with bland or hemorrhagic stroke [55]. Patients were 38 – 72 years old. The study was not randomized, blinded or placebo controlled. Forty two patients were treated daily with IV *fufang danshen *containing 100 mg of tanshinone IIA. Fifty three patients were treated daily with *fufang danshen *containing 50 mg of tanshinone IIA. The treatment period ranged from 1 to 4 weeks. Patients were assessed in terms of symptom improvement. A standard format of clinical assessment was adopted in China at this time. The treatment was described as a cure, very effective, effective, not effective or the treatment made symptoms worse. High dose treatments resulted in a 95% total effective rate. Low dose treatments were 79% totally effective. The total effective rate is derived by adding the numbers of patients from the cure, very effective and effective groups.

Thirty stroke patients were studied for quality of life improvements after stroke treatment with *fufang danshen *[56]. There were 20 male and 10 female patients of average age 64. The study was not randomized, blinded or placebo controlled. *Fufang danshen *was administered daily into the carotid artery, on the affected side, in doses that contained 10 mg of tanshinone IIA. Treatments started 2 to 77 days after stroke. Patients were assessed in terms of CT brain scans, paralysis, independent living and other quality of life measurements. The effective rate was 97%.

Fifty six patients suffering from angina or angina associated with previous myocardial infarction were studied in a 1997 article [57]. There were 38 male and 18 female patients aged 39 – 68. Thirty three patients had angina alone. Twenty three patients suffered from angina and had previous myocardial infarctions. All patients were evaluated in terms of clinical symptoms and ECG changes. The study was not randomized, blinded or placebo controlled. *Fufang danshen *containing *Dalbergia odorifera *was administered in IV doses containing 40 – 80 mg of tanshinone IIA. The drug was given daily over a period of 14 – 30 days. Standard drugs used in heart disease, such as dipyridamole, were not discontinued. *S. miltiorrhiza *was found to be 89% effective at improving angina paroxysms. It was 67% effective at improving conductance in the heart as indicated by ECG changes.

Cor pulmonale was examined in 52 patients [58]. There were 24 male and 28 female patients. The average age of the patients was 78. There were two treatment groups, *fufang danshen *and routine Western therapy. *Fufang danshen *was administered to 31 patients. The Western therapy group included 21 patients. The study was blinded but not randomized. *Fufang danshen *with *Dalbergia odorifera *was administered in IV doses containing 50 mg of tanshinone IIA daily for 7 – 10 days. Western therapy included cardiotonics, diuretics and other drugs. Patients were evaluated in terms of breathing, heart function, edema, arrhythmias and other symptoms. *S. miltiorrhiza *was found to be 87% effective. Western therapy was 62% effective. *S. miltiorrhiza *was significantly better than Western therapy.

A 1997 publication discussed several clinical trials of *S. miltiorrhiza *[59]. None of the trials appear to be randomized, blinded or placebo controlled. A study of subarachnoid cavity bleeding administered IV *S. miltiorrhiza *in doses containing 100 mg of tanshinone IIA to 24 patients daily for 10 days. Patients were also treated with anti-hypertensives and vasodilators as needed. High intracranial pressure was treated with IV infusions of 20% mannitol or 50% glucose 3 – 4 times a day for 3 – 5 days. Twenty three patients improved. One patient died. A study of 48 patients suffering from cerebral thrombosis was discussed. *S. miltiorrhiza *was administered daily in IV doses containing 75 mg of tanshinone IIA. The discussion indicated that *S. miltiorrhiza *was effective at reducing the sequelae of cerebral thrombosis. A study of angina patients compared pentaerythritol tetranitrate to the combination of *S. miltiorrhiza *and pentaerythritol tetranitrate. Patients were assessed for clinical improvement and ECG changes. The combination of *S. miltiorrhiza *and pentaerythritol tetranitrate was significantly better than pentaerythritol tetranitrate alone.

*S. miltiorrhiza *has been manufactured in several pill forms, such as composite *danshen *droplet pills [60]. The amount of tanshinone IIA in these pills is not published. A trial of these pills was randomized, blinded and placebo controlled. The *danshen *droplet pills were described as effective in the treatment of angina and more effective than placebo.

After 1997, many studies were published comparing *S. miltiorrhiza *to other herbal preparations. *S. miltiorrhiza *had become the standard therapy for stroke and other ischemic conditions in China. All new preparations had to be compared to *S. miltiorrhiza*. A 1998 study compared *S. miltiorrhiza *to a combination of *S. miltiorrhiza *and *huangqi *[61]. *Huangqi *is *Astragalus membranaceus *and is used to reestablish *qi. Astragalus membranaceus *contains several glycosides, including astragaloside IV that is neuroprotective in a stroke model [62,63]. The *S. miltiorrhiza *alone group contained 78 patients of average age 68. All patients had suffered from strokes 1 – 10 days before admission to the study. *S. miltiorrhiza *was given IV in doses containing 40 mg of tanshinone IIA twice a day for 28 days. The trial was not randomized, blinded or placebo controlled. Patients were assessed for hematocrit, blood viscosity and other parameters. *S. miltiorrhiza *was 69% effective. *Astragalus membranaceus *improved the efficacy of *S. miltiorrhiza*.

A later study re-evaluated *Astragalus membranaceus *and *S. miltiorrhiza *in stroke [64]. Twenty patients were treated with *S. miltiorrhiza *alone in IV doses containing 40 mg of tanshinone IIA daily for 20 days. The time of administration after stroke was not specified. The trial was not blinded, randomized or placebo controlled. Patients were evaluated for neurological status and plasma levels of prostaglandin I_2 _and thromboxane A_2_. *S. miltiorrhiza *provided a 75% improvement in clinical status, increased prostaglandin I_2 _levels and decreased thromboxane A_2 _levels. The combination of *Astragalus membranaceus *and *S. miltiorrhiza *was found to be superior to *S. miltiorrhiza *alone.

A 1999 publication compared *S. miltiorrhiza *to an unspecified anti-thrombotic enzyme [65]. Patients were older than 75, had suffered from strokes that impaired consciousness, had atrial fibrillation and a history of hemorrhage. The treatment group received the anti-thrombotic enzyme. The control group was administered IV *S. miltiorrhiza *containing 50 mg of tanshinone IIA. Drug administration occurred as soon as possible after stroke, from 6 to > 48 hours after stroke. The trial was not blinded, randomized or placebo controlled. *S. miltiorrhiza *alone, given within 6 hours after stroke, was 55% effective in terms of improving neurological functions. The anti-thrombotic enzyme was 90% effective when administered within 6 hours after a stroke.

*S. miltiorrhiza *was compared to naloxone in a trial of patients who remained unconscious after stroke [66]. The group that received *S. miltiorrhiza *alone included 35 patients of average age 61. There were 21 males and 14 females. *S. miltiorrhiza *or naloxone was administered as soon as possible after stroke in unspecified doses. The trial was not randomized, blinded or placebo controlled. *S. miltiorrhiza *was 74% effective at improving neurological functions. Patients regained consciousness in 36 ± 16 hours after admission to the study. Naloxone appeared to be more effective than *S. miltiorrhiza*.

A later study recompared naloxone and *S. miltiorrhiza *in stroke patients [67]. Ninety six stroke patients were randomly assigned to *S. miltiorrhiza *or naloxone groups. The study was not blinded or placebo controlled. *Fufang danshen *was administered in doses containing 40 mg of tanshinone IIA, daily for 10 days. Naloxone was given in doses of 2 mg per day for 10 days. Patients were assessed for improvement of neurological symptoms. The *S. miltiorrhiza *group demonstrated 58.3% improvement. Naloxone treated patients had a 92% improvement in symptoms.

*S. miltiorrhiza *was also compared to naloxone in heart attack patients [68]. The study was not randomized, blinded or placebo controlled. Seventy two patients were given either naloxone 0.8 g per day for 5 days or *fufang danshen *containing 50 mg of tanshinone IIA daily for 5 days. Symptoms and hemorrheological parameters were assessed. *S. miltiorrhiza *was 89% effective compared to 75% efficacy for naloxone. *Fufang danshen *decreased blood viscosity and fibrinogen content. The side effects of *fufang danshen *were nausea, vomiting and facial flushing.

Nicergoline, an ergot alkaloid, was compared to *S. miltiorrhiza *in patients suffering from acute stroke [69]. The trial was randomized, but not blinded or placebo controlled. *S. miltiorrhiza *alone was administered daily for 15 days in IV doses containing 50 mg of tanshinone IIA to 50 patients. The time of administration after stroke was not specified. Patients were assessed for neurological status and cerebral blood flow. *S. miltiorrhiza *was 72% effective at improving neurological status and improved cerebral blood flow to some extent. The combination of nicergoline and S. miltiorrhiza was reported to be superior to *S. miltiorrhiza *alone.

Prostaglandin E_1 _was compared to *S. miltiorrhiza *in a trial of acute stroke patients [70]. Patients were treated soon after stroke with IV prostaglandin E_1 _(200 μg) or IV *S. miltiorrhiza *containing 40 mg of tanshinone IIA. *S. miltiorrhiza *was administered daily for 15 days. The trial was not randomized, blinded or placebo controlled. *S. miltiorrhiza *was 90% effective at improving neurological status, decreased blood viscosity, cholesterol and triglycerides. Prostaglandin E_1 _treatment was as effective as *S. miltiorrhiza *treatment.

Several trials have compared *S. miltiorrhiza *to other Chinese herbal medicines, such as *honghuayou *and *gegen. Honghua *is *Carthamus tinctorius *also known as safflower. Safflower oil, *honghuayou *in Chinese, is used in the treatment of ischemic conditions. *Gegen *is *Pueraria lobata*. A comparison of *honghua *and *S. miltiorrhiza *was performed in 60 patients in randomly assigned groups [71]. The study was not blinded or placebo controlled. Patients were treated within 72 hours of stroke. The *Carthamus tinctorius *oil group received 20 ml of safflower oil in 500 ml of 5% glucose solution, daily. The *fufang danshen *group received daily doses containing 50 mg of tanshinone IIA in a solution of low molecular weight glycogen. There were 30 patients, 18 males and 12 females, of average age 59 in the *gegen *group. The *fufang danshen *group had 17 male and 13 female patients of average age 59. Patients in both groups were treated for 14 days and evaluated for neurological symptoms. The *Carthamus tinctorius *effective rate was 93% compared to 90% for *fufang danshen*. Another stroke study [72] of *Carthamus tinctorius *oil, 40 ml, and *S. miltiorrhiza*, 40 mg of tanshinone IIA, examined 36 *Carthamus tinctorius *oil patients and 32 *S. miltiorrhiza *patients. The study was not blinded, randomized or placebo controlled. *Carthamus tinctorius *was 95% effective and significantly more effective than *S. miltiorrhiza*, 84%. Both groups experienced reduced blood viscosity and blood fat. A *Pueraria lobata *study in 60 stroke patients administered 500 mg of *gegensu *(*Pueraria lobata *factor) to 18 male and 12 female patients [73]. *Fufang danshen *was administered in doses containing 50 mg of tanshinone IIA to 19 male and 11 female patients. The study was not randomized, blinded or placebo controlled. Patients were treated daily for 14 days then evaluated for neurological symptoms and by CAT scan. *Pueraria lobata *was 70% effective compared to 33% for *S. miltiorrhiza*.

*S. miltiorrhiza *has been compared to oxygen in the treatment of 102 stroke patients [74]. The study was not blinded, randomized or placebo controlled. Patients had suffered from strokes 30 minutes to 48 hours prior to being admitted to the study. CAT scan analysis showed blocked blood flow in the brains of the patients. *S. miltiorrhiza *was administered IV to 34 male and 18 female patients of average age 58 in doses containing 50 mg of tanshinone IIA. This group of patients also received oxygen by face mask for 1 hour daily for 10 days. The second group of 31 male and 19 female patients of average age 57 received only *S. miltiorrhiza*. Patients were assessed for neurological symptoms, blood flow, blood viscosity, clotting and sedimentation rate 10 days after admission. Both therapies were 100% effective. *S. miltiorrhiza *with oxygen produced a cure rate of 23%, in other words no residual neurological symptoms. *S. miltiorrhiza *alone produced a cure rate of 18%.

Acuthrombin-B, an enzyme with thrombin-like activity from the venom of *Deinagkistrodon acutus *the hundred pace pit viper, has been compared to *S. miltiorrhiza *in stroke patients [75]. The study was not randomized, blinded or placebo controlled. Sixty two patients, 38 males and 24 females of average age 62, were IV administered 0.75 U of snake enzyme daily for 7 days. The *fufang danshen *group of 39 males and 23 females of average age 63, were given IV *S. miltiorrhiza *containing 40 mg of tanshinone IIA daily for 14 days. At 14 days the patients were assessed for neurological symptoms. The total effective rate for acuthrombin-B was 85% compared to 68% for *S. miltiorrhiza*.

A study of patients undergoing cardiopulmonary bypass procedures examined the effects of *S. miltiorrhiza *on endothelin, prostaglandins and thromboxanes [76]. Endothelin is a vasoconstrictor peptide found in the body. The study was not randomized or blinded. The treatment group consisted of 10 patients treated with 200 mg/kg of tanshinone IIA immediately after surgery. The placebo control group of 10 patients received saline. Endothelin decreased while prostaglandin I_2 _(PGI_2_) and thromboxane A_2 _(TXA_2_) increased in both groups during surgery. Endothelin increased from 30 minutes to 24 hours after reperfusion. The increase in endothelin was less in the tanshinone group than the placebo group. PGI_2 _and TXA_2 _decreased during reperfusion. The tanshinone group had a more rapid decrease in these factors while the ratio of PGI_2_/TXA_2 _was higher than the placebo group. These factors are involved in control of vascular tone and platelet aggregation that are critical to patients suffering from ischemic conditions.

Buflomedil, an α_1A _receptor blocking vasodilator, has been compared to *S. miltiorrhiza *in stroke patients, without intra-cerebral hemorrhage [77]. The patients were randomly assigned to buflomedil or *S. miltiorrhiza *groups. The study was not blinded or placebo controlled. Buflomedil, 200–300 mg, or *fufang danshen*, containing 50 mg of tanshinone IIA were administered daily for 14 days. The buflomedil group contained 30 males and 12 females of average age 58. The *S. miltiorrhiza *group contained 32 males and 13 females of average age 56. On day 15, patients were evaluated for neurological symptoms. Buflomedil was 90% effective. *S. miltiorrhiza *was 69% effective and significantly decreased blood viscosity.

A study of 100 angina patients compared two pill forms of *S. miltiorrhiza*, Coronary Heart *Radix Salvia Miltiorrhiza *Drop Pill and Coronary Heart *Radix Salvia Miltiorrhiza *Tablet [78]. The study was blinded and randomized but not placebo controlled. Both pills were administered sublingually. The drop pill contained 0.065 mg of salvianolic acid B per pill and was used in doses of 10 pills 3 times daily, except during angina attacks when 15–20 pills were used. The tablet contained an unspecified amount of tanshinone IIA and was used in doses of 3 pills 3 times daily, except during angina attacks when the dose increased to 4–5 pills. Both pills improved chest pain, ECG changes and decreased blood viscosity. The drop pill was 93% effective. The tablet was 88% effective.

Carnitine was compared to *S. miltiorrhiza *in a study of 135 patients [79]. The study was not randomized, blinded or placebo controlled. Patients had suffered from strokes within 7 days of admission to the study. Thirty seven male and 31 female patients were administered 1 g of carnitine IV daily for 28 days. Thirty nine male and 28 female patients were given IV *fufang danshen *containing 50 mg of tanshinone IIA daily for 28 days. Carnitine produced a 99% effective rate, while *S. miltiorrhiza *was 65% effective at improving neurological symptoms.

A study of *S. miltiorrhiza *in 68 stroke patients administered composite salvia injection in unspecified doses [80]. The patients were randomly divided into groups that received either *S. miltiorrhiza *or a control medicine referred to as *xueshuantong *(embolism medicine). The total effective rate of *S. miltiorrhiza *was 88%. The placebo effective rate was not reported.

Tanshinone IIA was administered in daily doses of 1 g to patients who had suffered strokes from 1 month to 2 years previously [81]. All patients had persistent symptoms of stroke such as paralysis, low muscle strength, movement problems, speech difficulty and memory impairment. Tanshinone IIA was compared to yang 5 ingredient soup. The decoction contains *Angelica sinensis*, *Prunus persica*, *Tagetes erecta*, *Ligusticum wallichii*, *S. miltiorrhiza*, *Paeonia lactiflora*, *Pherectima aspergillum *and *Astragalus membranaceus*. The study was not blinded, randomized or placebo controlled. Tanshinone IIA was given 3 times daily IV for 4 weeks. Yang 5 ingredient decoction was given orally, 200 ml, twice daily for 4 weeks. Patients were then assessed for muscle strength in the arms, legs and abdomen. Tanshinone IIA was 96% effective and cured 21% of patients. Yang 5 ingredient decoction was 59% effective and cured 12% of patients of stroke symptoms.

Pregnancy induced hypertension was investigated in 60 patients [82]. The study was not blinded, randomized or placebo controlled. *S. miltiorrhiza *injection containing 40 mg of tanshinone IIA was administered daily for 10 days. *S. miltiorrhiza *decreased blood viscosity, cholesterol and lipoprotein levels.

A study of 120 stroke patients compared *S. miltiorrhiza *and defibrase, which was not identified but may be a snake venom fibrin cleaving enzyme [83]. The study was not randomized or blinded. *S. miltiorrhiza *was used as the control. *S. miltiorrhiza *containing 50 mg of tanshinone IIA was administered IV daily for 14 days to one third of the patients. The effective rate of *S. miltiorrhiza *was 78%. *S. miltiorrhiza *also improved neurological symptoms 3 months after administration but did not alter carotid artery atherosclerosis.

Percutaneous coronary intervention and the effects of *fufang danshen *were studied in 38 myocardial infarction patients [84]. The study was randomized but not blinded. Control patients received no *fufang danshen*. This *fufang danshen *contained *Panax ginseng, Schisandra *berry and *Ophiopogonis *as well as *S. miltiorrhiza*. All patients received percutaneous coronary intervention. Left ventricular function and various indications of oxidative damage improved more in the *fufang danshen *group than the control group.

Blood coagulation was investigated in 64 traumatic brain injury patients [85]. The study was randomized, not blinded but was placebo controlled. The treatment group received an unspecified dose of *S. miltiorrhiza *as well as conventional Western medicine. The placebo group received conventional Western medicine. *S. miltiorrhiza *improved the neurological status of patients as well as blood coagulation factors such as plasma P selectin, von Willebrand's factor and D dimer.

### Limitations of the trials

Time of treatment after stroke, dose, type of *fufang*, randomized design, placebo controls and difficulty in finding the published trials are some of the problems encountered with these trials. Time of treatment after onset of ischemia varies from a few hours to many days. Yet improvements have been noted in several studies when *S. miltiorrhiza *was started many days after an ischemic event such as a stroke. These results imply that *S. miltiorrhiza *may stimulate brain repair mechanisms, perhaps by stimulating stem cell growth, neurotrophin release or other mechanisms. The dose of tanshinone IIA has been found and reported above for most of the trials. However, some of the trials did not give enough information to allow calculation of tanshinone IIA doses. The *fufang danshen *used in published trials is usually the type containing *Dalbergia odorifera*. However, some trials did not give enough information to allow identification of the type of *fufang *used. Few of the published trials were randomized or blinded. This makes bias in patient selection a possibility. Few of the published trials were placebo controlled. This makes comparison impossible between the effects of *S. miltiorrhiza *and those of other therapies in stroke. After *S. miltiorrhiza *was accepted as the standard for the treatment of stroke, *S. miltiorrhiza *became the control for other trials.

It is difficult, in the USA, to obtain copies of some of the published trials. The current study found publications for 33 clinical trials of *S. miltiorrhiza*. There are many more trials referred to in the Chinese literature but not easily accessable. The open exchange of the information about clinical trials should be improved.

## Meta-analyses

Meta-analyses help justify the collection of data from different studies into one analysis. Three meta-analyses of *S. miltiorrhiza *have been published [1,45,46]. Two of the meta-analyses found that insufficient evidence has been published from randomized, blinded, placebo controlled trials to support the efficacy of *S. miltiorrhiza *in stroke. The other meta-analysis found that there was no evidence to suggest that *S. miltiorrhiza *is effective against the disability caused by stroke. Meta-analysis cannot be conclusive as there is a huge variation in the design of clinical trials.

Given the discussion presented above of 33 clinical trials of *S. miltiorrhiza*, it is obvious that few of the studies are similar enough to justify collecting all their data into one analysis. However, *S. miltiorrhiza *has been found to benefit stroke patients, as well as angina and other patients, in every published trial. There is a great danger of ignoring an obvious effect of *S. miltiorrhiza *when meta analysis cannot be used successfully. Since 1997, when *S. miltiorrhiza *became the common medication for stroke in China, 17 reports of clinical trials have been published on the use of *S. miltiorrhiza *in stroke. All these trials found that *S. miltiorrhiza *improved quality of life in stroke patients. *S. miltiorrhiza *was found to be effective at decreasing the neurological symptoms of stroke in a range of 33% – 100%, with a mean of 74%. It may be a mistake to assert that *S. miltiorrhiza *has no efficacy in treating stroke.

## Discussion

The main approach of Western medicine is preventing stroke. There is sometimes an effort made by primary care physicians to encourage patients to lose weight and exercise as preventive medicine against stroke. Anticoagulants and aspirin are sometimes recommended to prevent clots that may cause strokes. In the USA, stroke is treated with tissue plasminogen activator 3 hours after stroke in a minority of eligible patients. The remaining, ineligible patients are usually not attended to until 6 hours after stroke. This is when the neurological scores tend to stabilize, as the stroke damage matures in the brain. However, other than tissue plasminogen activator, there is no effort to treat stroke patients with drugs that might enhance their survival or recovery.

The herbal approach to stroke treatment is to find a plant or combination of plants that helps patients survive and recover from stroke. The drug approach involves finding a single drug and a single mechanism to treat stroke. This is driven by the US Food and Drug Administration requirements for drug approval. However, this single minded approach may explain why no effective treatment of stroke exists in the USA. It may be that multiple mechanisms must be exploited simultaneously in order to ameliorate stroke symptoms. There is evidence in a stroke model that using drugs with two different mechanisms together can enhance stroke survival and recovery [86].

## Conclusion

*S. miltiorrhiza *is a very useful herb in the treatment of cardiovascular disorders. It has been extensively studied in clinical trials in China. Most of the clinical trials have some limitations. However, taken as a whole, the clinical trials present compelling evidence of the efficacy of *S. miltiorrhiza *in stroke, heart attack and other conditions. Further research is needed to understand all actions of *S. miltiorrhiza*, and all active compounds found in the plant, in treating cardiovascular diseases. Research is also needed to help us understand the pharmacological effects of other herbs added to *S. miltiorrhiza*.

## Competing interests

The author(s) declare that they have no competing interests.

## Authors' contributions

JA compiled all data and clinical trial publications, discussed the data with all the authors, wrote and edited the manuscript. RW helped to collect some of the publications of clinical trials, translated all articles from Chinese to English, discussed all data and edited the manuscript. JY helped to collect some of the publications of clinical trials, discussed all data, and edited the manuscript especially in the area of Traditional Chinese Medicine. EL helped to compile some of the chemistry data, helped with the interpretation of Chinese to English, discussed all data, and edited the manuscript.
